# On the Comparison Between the Nc/CRN and the Ne/ERN

**DOI:** 10.3389/fnhum.2021.788167

**Published:** 2022-06-23

**Authors:** Franck Vidal, Boris Burle, Thierry Hasbroucq

**Affiliations:** Aix-Marseille Université, CNRS, LNC UMR 7291, Marseille, France

**Keywords:** Nc/CRN, Ne/ERN, errors, anterior cingulate cortex, supplementary motor areas, action monitoring

## Abstract

After the Error Negativity (Ne or ERN) has been described on full-blown errors and on partial error, a smaller Error Negativity-like wave (CRN or Nc) has also been evidenced on correct trials, first in patients with schizophrenia and, later on, in healthy subjects. The functional significance of the Nc as compared to the Ne is of critical importance since most models accounting for the genesis of the Ne on errors and partial errors cannot account for the existence of the Nc if this Nc simply corresponds to a small Ne. On the contrary, if the Nc and the Ne are two completely distinct components, then the existence of a Nc poses no constraint to the existing models. To this end, we examine in the present review the similarities and the differences existing between the Ne and the Nc regarding their functional properties and their anatomical origin.

## Introduction

The study of error processing in speeded choice conditions dates back to the 1960s with the pioneering work of Rabbitt (1966), and resorted to the field of Experimental Psychology. In the early 1990s, the discovery of the Error Negativity (Ne) in choice reaction time (RT) paradigms by Falkenstein et al. (1991), confirmed shortly after by Gehring et al. (1993: Error-Related Negativity or ERN), introduced error processing studies in the field of Neuroscience where they went on prospering up to now.

The Ne was evidenced as a large response-locked event-related potential (ERP), peaking about 100 ms after erroneous responses committed in choice or Go/No-go RT conditions with maximum amplitude at midline frontocentral sites. Its onset preceded the mechanical erroneous response (Falkenstein et al., 1991), but shortly followed erroneous electromyography (EMG) onset (Gehring et al., 1993). Initially, no response-locked negative component could be evidenced on correct responses; therefore, the Ne has been considered to be specific to errors (e.g., Falkenstein et al., 1991; Gehring et al., 1993; Coles et al., 2001). Scheffers et al. (1996) demonstrated that incorrect subthreshold EMG activations not strong enough to trigger an overt erroneous response (called partial errors), followed by a correct response in the same trial, also evoked a Ne which amplitude was smaller than that evoked by full-blown errors. Later on, Ford (1999) reported that correct responses evoked the same negative component as errors in patients with schizophrenia (Correct-Related Negativity or CRN: Ford, 1999, also called Nc: Yordanova et al., 2004) but not in healthy control subjects realizing the same task, which was considered as evidence that patients could not distinguish their correct responses from their errors. Finally, Vidal et al. (2000), thanks to the use of the Laplacian transformation (Hjorth, 1975) unmasked an Nc on correct responses, smaller than the Ne in healthy subjects, which had remained unnoticed so far because it was overlapped by larger components issued from remote generators.^1^ Although this Nc might have been generated by (1) “false” error detections or (2) contamination by stimulus-locked ERPs on very fast RTs (Coles et al., 2001), these possibilities have been discarded because (1) the Nc was present with the same amplitude in simple and choice RT conditions (Vidal et al., 2000) and (2) it could be demonstrated that the Nc was response-locked on all the distribution of correct RTs (Vidal et al., 2003). Now, the functional significance of the Nc in healthy subjects is still not clearly settled yet; is the Nc a small Ne or does it correspond to a different component? To answer this question, it is necessary to compare their respective properties. This is the aim of the present article.

## Why Is the Question of Same *vs.* Different Components Relevant?

Before asking whether the Ne and the Nc represent a same component and discuss about the relevance of this question, the notion of what a component is, or should be considered as, is to be shortly presented.

### What Does One Mean by “Component”?

Whatever the definition given to the term component, the study of components aims at identifying electroencephalographic indices of identifiable mental processes. To do so, the corresponding brain activities must be isolated from other ones both in space and time.

A frequent characterization of an ERP component is simply descriptive: a component is defined as a deflection of a given polarity, with maximum at a given scalp location, in a given latency range, in given classes of experimental conditions. With such a factual definition, nothing warrants that a component is an index of one single identifiable mental process, uncontaminated by other distinct mental processes. These so-called components might simply result from a weighted summation of several brain activities corresponding to separate mental processes developing in similar latency ranges, and which overlap in time and space. Moreover, given this definition, several characteristics of a component would depend on the chosen recording reference.

For these reasons, following Donchin et al. (1978), we consider that such a definition is disadvantageous and we will rather adopt the following one proposed by several authors. This definition, instead of being descriptive, is (psycho) physiological and suitable to identify separately mental processes, as illustrated by Tenke and Kayser (2005). According to these authors, a component would correspond to a brain activity with “…an identifiable neuroanatomical origin…having a distinct time course…that can be shown to vary as function of experimental manipulation” (page 2830; see also Kayser and Tenke, 2005). A similar conception can be found in a very influential guidelines article by Picton et al. (2000): “Ultimately, the goal is to understand the ERP waveforms in terms of both intracerebral sources and experimental manipulations. A component would then be a temporal pattern of activity in a particular region of the brain that relates in a specific way to how the brain processes information” (page 141).

We will henceforth use this acceptation when referring to a component. Otherwise, we will use the factual term of “wave.”

It must be pointed out that a difficulty immediately arises with such a definition: identifying separately the activities issuing from different brain structures. Note, however, that separating the activities issuing from different brain structures or separately identifying different components does not necessarily require localizing their generators (see Burle et al., 2015 for a detailed discussion on the separation issue).

### Methodological Consequences

If one concludes that the Ne and the Nc correspond to different components, then “errors do not simply amplify monitoring processes commonly engaged during both correct and incorrect actions, but rather, activate a specialized system for the detection of and adjustment to errors” (Vocat et al., 2008, page 2,554). This conception suggests that on errors and on errors only (whether partial or full-blown), a specific “true” Ne probably adds to the Nc which might represent some kind of mandatory motor-like component on which the true Ne would superimpose on errors.

A logical consequence is to assimilate the true Ne as this additional activity evoked on errors and on errors only. Considering this activity as additional on errors allows identifying it as the difference between ERPs obtained on errors and on correct responses (provided that the two components have the same latency which is not exactly the case).^2^ This has been clearly stated, for example, by Torpey et al. (2012); “…brain activity on error trials might reflect a combination of error-specific activity and activity that occurs on all trials due to button presses. Subtracting correct from error trials…is an attempt to isolate neural activity specific to errors” (page 147). This is certainly why, in search for the localization of the generator of the Ne, several authors choose to localize and/or examine the scalp distribution of the difference waveform between correct and error trials (e.g., Alain et al., 2002; Vocat et al., 2008; Reinhart et al., 2012). Note that if the Ne and the Nc correspond to a same component, this approach is invalid.

In this line of reasoning, relying on the difference waveform only could also be misleading.

For example, Ford (1999) reported that in patients with schizophrenia, the Ne is decreased on errors while the Nc is increased (see also Alain et al., 2002; Mathalon et al., 2002) to such a point that both waves have the same amplitude. In patients with prefrontal lesions, the Ne and the Nc are also of equal size (Gehring and Knight, 2000; Hogan et al., 2006). In these cases, a difference waveform would be of null amplitude (e.g., Figure 1 of Alain et al., 2002) and would lead one to conclude that (electro) physiological processes underlying action monitoring disappeared in these patients. However, these processes are still present and active, although not functional. In patients with Alzheimer’s disease, a difference wave would also yield a null amplitude but for a very different reason: there is no identifiable negative wave at the Ne or Nc latencies, neither on errors nor on correct trials (Mathalon et al., 2003). Therefore, contrary to patients with schizophrenia or patients with prefrontal lesions, in patients with Alzheimer’s disease, the physiological processes underlying action monitoring are not functional because these are not active anymore.

**FIGURE 1:**
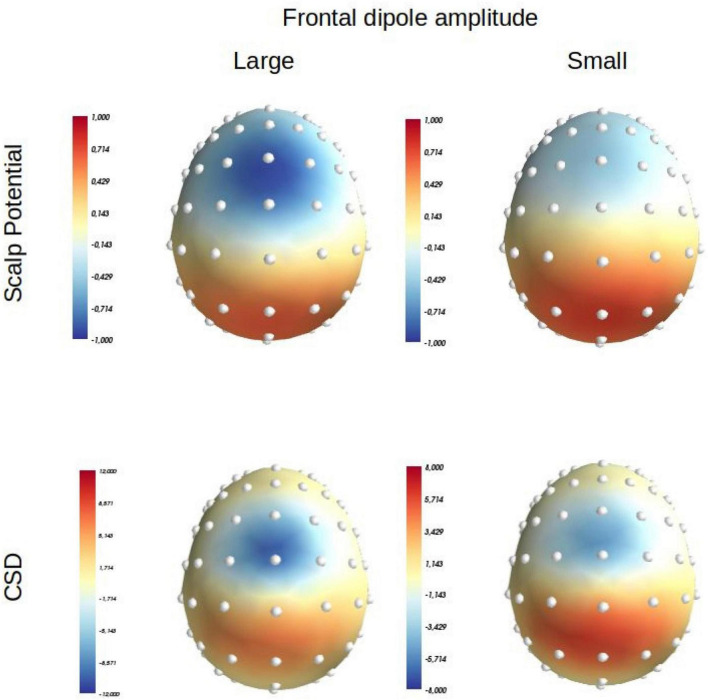
Scalp potential (average reference) and Laplacian (or Current Source Density: CSD) distributions produced at scalp level by a positive medial parietal dipolar source positioned at: *x* = 0; *y* = –30, *z* = 35 (orientation: *x* = 0, *y* = –0.4, *z* = 0.4) in MNI coordinates, and a medial frontal source positioned at: *x* = 0, *y* = 0, *z* = 50 (orientation: *x* = 0, *y* = 0.1, *z* = 1) in MNI coordinates. Left panel: the intensity of the parietalsource is set at 40 mA/m3 and that of the frontal one at –20 mA/m3. Right, panel: the intensity of the parietal source is set at 40 mA/m3 and that of the frontal one at –10 mA/m3 [adapted from Vidal et al. (2015)].

Now, one could argue that these examples are from patients, and that the recruitment of the different generators at work on correct responses and on errors in patients cannot be extrapolated from the normal processes at work in healthy subjects. Meckler et al. (2011) submitted healthy subjects to a biased between-hand two-choice RT task: 80% of the responses were to be given with one hand (expected condition) and 20% had to be given with the other hand (unexpected condition). In the expected condition, the Nc amplitude was identical to that elicited in the same subjects in a more classical right 50%/left 50% condition. On the contrary, in the unexpected condition, the Ne was slightly (but significantly) reduced while the Nc was largely enhanced to such a point that the small remaining difference between the Nc and the Ne (although probably still present) did not reach the significance level. If the Nc and the Ne are two different components, then it could seem appropriate to rely on the difference between error and correct trials to understand the effect of expectancy in healthy subjects. The conclusion would then be that the action monitoring system as revealed by the (true) Ne in this latency range cannot work anymore on unexpected trials. On the contrary, if one considers that the Ne and the Nc are a same component, one would conclude that the action monitoring system does work on correct and error trials but loses its sensitivity to the context on unexpected trials in this latency range.

An *ad hoc* explanation of this dataset preserving the idea that the Nc and the Ne are different components could consist in assuming that in unexpected trials, subjects committed “false” error detections on correct responses and that a true Ne was superimposed on the “regular” Nc in this particular class of correct trials. In this case, the subtraction method would be inappropriate because response-locked ERPs of these special correct trials would also contain a Ne. But on which criterion or experimental argument one (*a priori*) decides that a Nc of a given condition should be “pure” but contaminated in another condition by a superimposed Ne due to assumed false error detections?

Now, when subjects have to overtly rate their responses as correct or erroneous, relying on the difference between correct and error ERPs could lead to quite counterintuitive conclusion which cannot be accounted for by the idea that enhanced Nc reflects the superimposition of a Ne due to false error detections. Grützmann et al. (2014) compared the Ne and the Nc in subjects who performed a flanker task in two conditions: a non-rating condition and a rating condition in which subjects had to decide, on each trial, whether their response was correct or erroneous. In the rating condition, both the Ne and the Nc were enhanced as compared to the non-rating one. However, the difference between the Ne and the Nc remained unchanged across conditions. Whatever the exact reason for the effect of this manipulation, relying on the difference between the Ne and the Nc would counterintuitively lead to conclude that the (true) Ne is insensitive to rating (which nevertheless could be possible). In this case, the enhancement of the Ne wave on errors should simply be explained by an increase of the Nc which, being mandatory on correct and errors, would also be present and increased on errors. However, how to explain the increase of the Nc when overt evaluation is required? An explanation in terms of false error detections on correct trials in the rating condition does not hold: the proportion of misclassification of correct responses was negligible: 1% on compatible and incompatible trials (“unsure” responses were also very scarce: compatible: 1%, incompatible: 3%). It is therefore hard to imagine that the action monitoring system did better at classifying the responses in the non-rating condition. Thus, increase of false error detections on correct trials hardly accounts for the increased Nc on correct trials in the rating condition. Now, if the Ne and the Nc correspond to a same component, one would simply conclude that the rating manipulation uniformly increased the action monitoring process subserved by this component on correct and on error trials.

Models, and the interpretations they imply, can also be affected by the subtracting method. For example, in the presentation of the reinforcement learning model (Holroyd and Coles, 2002), the relevant comparison between the output of the simulations and ERPs of human subjects is on different waves between correct and error trials (probably because, as we will see below, the model assumes no output signal on correct trials). Therefore, the effect of simulated or real experimental manipulations (such as frequency or compatibility in Holroyd and Coles, 2002) might be due either to the Ne, to the Nc, or even both (whether or not the Ne and the Nc are a same component). As a consequence, it may be quite hard to understand what exactly do the results of a simulation model when relying on difference waves.

### Constraints That May (or May Not) Be Imposed to the Models

Several models have been proposed to account for the elicitation of the Ne, the main ones being the error detection or mismatch model, the conflict model, the reinforcement learning model, the predicted response-outcome model and the reward value and prediction model.

#### The Mismatch Theory

From the very beginning, the apparent specificity of the response-locked negative wave observed on errors led the pioneering authors to consider it a physiological marker of an error detection process (Falkenstein et al., 1991; Gehring et al., 1993). This idea has been somewhat more formalized by Coles et al. (2001) with the mismatch model. According to this model, given that in most of the tasks used to study action monitoring, there is no ambiguity regarding either the nature of the presented stimuli or the stimulus-to-response rule of association, most errors result from premature decisions occurring while stimulus evaluation is still incomplete. At the moment when the incorrect response is activated, stimulus processing is still ongoing. The structure(s) that elicit the Ne would act as a comparator between (1) a representation of the required responses established on the basis of (still ongoing) stimulus processing and (2) a representation of the actually activated response established on the basis of an efference copy (Gehring et al., 1993). On errors, the output of the structure(s) comparing both representations generates a mismatch which would elicit the Ne as an error signal. On correct trials, there is no mismatch and, therefore, no Ne is expected. The smaller Ne on partial errors fits quite well with this model because partial errors can be considered as small errors which generate smaller mismatches and smaller Ne. However, there is no way to explain the presence of a Ne (even smaller than on errors or partial errors) on correct trials because there should be no mismatch between the required and the ongoing response.

If the Nc is a different component than the Ne, then the existence of the Nc does not challenge the mismatch model. On the contrary, if the Nc and the Ne are a same component which varies in amplitude, then it is hard to admit that it represents a mismatch (e.g., Falkenstein et al., 2000; Vidal et al., 2000; Gehring et al., 2012).

#### The Conflict Model

Another prominent account for the elicitation of the Ne is the conflict model. This strictly formalized model is very attractive because it proposes that the action monitoring system relies on a very elementary and elegant process that does not require any structure to “know” that an error is occurring to generate an “alarm” signal on errors, warning that something is wrong.

According to the model (Carter et al., 1998; Botvinick et al., 2001, 2004), several mutually exclusive responses which inhibit each other may be concurrently activated to different extents and will therefore conflict – conflict being defined as the sum of the product of the activations of the possible responses weighted by the strengths of their inhibitory connections (Botvinick et al., 2001). The model shows that the large amplitude of the conflict and its time course on errors look very much like the large amplitude of the Ne and its time course (Botvinick et al., 2001; Yeung et al., 2004). Moreover, the conflict, as defined by the model, is sensitive to various experimental manipulations in a very same way as the Ne is.

In simulations, the conflict is especially large on errors but may also be present on correct trials. Therefore, at face value, the conflict model seems to easily account for the Nc as a small Ne on correct trials. However, simulations of the conflict time course indicate that although the maximum conflict should occur *after* the response on errors, it should take place *before* on correct trials (Yeung et al., 2004). Therefore, in its canonical implementation, the conflict model cannot accommodate the existence of the Nc if this wave corresponds to a small Ne because it peaks too late. On the contrary, if the Ne and the Nc are two different components, the existence of the Nc does not challenge the model.

#### The Reinforcement Learning Model

Let us now examine the influential reinforcement learning theory (Holroyd and Coles, 2002). This theory is also very well formalized in a computational model which makes accurate quantitative predictions. A core assumption of the model is “…that the ERN is elicited when a neural system first detects that the consequences of an action are worse than expected” (page 680), which should obviously be the case on errors. This signal is used by the motor system to optimize its responses in the ongoing task.

Although this model has been designed to account for the elicitation of the Ne, it predicts, as a side effect, that “The ERN is generated on error trials, but not on correct trials …” (page 686). Therefore, the model can accommodate the existence of the Nc if and only if the Ne and the Nc correspond to different components. If the Nc is a small Ne, then again, its presence challenges the model. Moreover, the simulations (assuming no output signal on correct trials) model the ERN as the difference wave between correct and errors, which is compared to the same difference wave in human subjects performing a task. If the Nc were a small Ne, then it would be hard to understand what exactly do the simulations model.

#### The Predicted Response-Outcome Model

As the preceding model, the predicted response-outcome model (Alexander and Brown, 2011) relies on a reinforcement learning law but, instead, it proposes that the Ne is evoked on errors not because they correspond to a bad outcome (worse than expected) but rather because this outcome is unexpected (unpredicted). More specifically “…the model specifically detects…when an expected event fails to occur (whether good or bad)…’’ (page 1,339). Given that, in most experimental contexts, errors are rare, the reinforcement learning model would correctly account for several experimental data just because unexpectedness and badness are confounded factors in these experiments. Therefore, in a given task context, any large prediction error (corresponding to an unexpected outcome)^3^ should evoke a large Ne/Nc whether on error or on correct trials. A logical consequence is that large unexpectedness signals should show up on correct trials when correct responses are not that frequent (not that expected) and errors are not that rare (not that unexpected).

Such a situation is observed in Meckler et al. (2011) data where in the unexpected condition, the amplitude of the Nc is increased while that of the Ne is reduced. If the Nc is a true Ne, these results seem to be predicted by the model. This is not the case if the Ne and the Nc correspond to separate components.

#### The Reward Value and Prediction Model

Still owing to a reinforcement learning law, sharing several properties with the preceding model, developed independently, the reward value and prediction model (Silvetti et al., 2011) predicts, *inter alia*, that the amplitudes and time courses of the Ne and of the Nc result from the computation of a prediction error, whether on correct (in the case of Nc) or on error (in the case of the Ne) trials. The reward value and prediction model states that “Error effects occur when error trials are less frequent than correct trials so that when an error occurs, a negative prediction error is detected” (see footnote) by an exponent 3 (page 9). Therefore, it is because in most experimental situations, errors are rare as compared to correct responses (and only because of their rareness) that they are unpredicted and, as a consequence, that the Ne is usually larger than the Nc in the predicted response-outcome model. The model states that this should not always be the case, which is in accordance with the results obtained by Meckler et al. (2011). Later on, this model has specifically been put to the test and received empirical support (Silvetti et al., 2014).

Finally, given that the model states that (1) a same process (computation of prediction error) generates the Ne *and* the Nc, (2) their time courses are supposed to be similar, and (3) they are generated by a same and unique structure in the medial prefrontal cortex, the model assumes that the Ne and the Nc correspond to a same component.

From this brief overview, one can see that some models would be (in)validated or not depending on the unitary nature of the Ne and the Nc.

Moreover, other than the consequences that this question of the same *vs.* different nature of the Ne and Nc may have on methodological of modeling issues, this question also has clear consequences for a better understanding of action monitoring processes, the comprehension of which being of importance for a more general human factors point of view (see Vidal et al., 2020 for the relevance of action monitoring to human factors studies).

## Comparison of the Functional Properties of the Ne and the Nc

### Main Differences

The Ne and the Nc have (slightly) different time courses (different peak latencies), different distributions of the surface potential (interpreted as reflecting different generators), and are elicited in qualitatively different experimental conditions (errors *vs.* correct trials). According to the definition proposed earlier of what a component is, each of these main differences between these two waves can be taken as a good argument in favor of the idea according to which the Ne and the Nc are different components. We will examine each of these points in the following sections.

#### Differences in Experimental Conditions of Elicitation

The first obvious difference between the Ne and the Nc corresponds to the experimental conditions in which these waves are elicited: errors for the Ne *vs.* correct responses for the Nc. However, the idea that these experimental conditions are different rests on (implicit) assumptions regarding the nature of the Ne. For example, in the predicted response-outcome model or in the reward value and prediction outcome model, the Ne would signal unexpected outcomes. According to this model, as regards the Ne, the difference between errors and correct trials is just quantitative, not qualitative. In most experimental contexts, correct outcomes are just more expected than are errors. However, in specific conditions, the difference of amplitude of the response-locked activities elicited on correct and error trials can almost vanish (Meckler et al., 2011) or could even be reversed (Alexander and Brown, 2011; Silvetti et al., 2011). Then, the difference between the conditions which elicit each wave may be quite relative and, overall, the assumed difference is model-dependent. Therefore, the contrast between error *vs.* correct conditions cannot constitute a definite argument to conclude that the Ne and the Nc correspond to different components.

#### Differences in Time Courses

Although the Ne and the Nc occur in the same latency range, the Nc and the Ne peak at different latencies, with the Nc peaking quite systematically earlier than the Ne on errors (e.g., Roger et al., 2010; Meckler et al., 2017). This difference in time courses might constitute an argument to consider that these two waves correspond to two different components.

However, it has been well established that the Ne on partial errors peaks earlier than the Ne on errors (e.g., Carbonnell and Falkenstein, 2006; Masaki et al., 2012).

Now, the latency of the Nc lies just in between that of the Ne on partial errors (later) and that of the Ne on errors (earlier) (Roger et al., 2010; Meckler et al., 2017). Therefore, these differences in latencies cannot argue in favor of the idea that the Ne and the Nc are different components.

#### Differences in Scalp Distribution

On surface potential recordings, the Ne and the Nc clearly present different distributions (Vocat et al., 2008; Meckler et al., 2011; Endrass et al., 2012; Grützmann et al., 2014; Files et al., 2021). Vocat et al. (2008) studied very finely the distributions of the Ne and the Nc. On the basis of the topographic differences that they identified in the distribution of the Ne and the Nc, these authors concluded that “although correct responses also elicited an early negative deflection…the topographic analyses revealed that the latter component had not only a smaller amplitude than the ERN/Ne, but more critically also a significantly different distribution… Such difference in topography is usually evidence that intracranial generators implicate distinct brain sources…” (page 2,554). In other words, still according to the definition of what a component is, adopted earlier, this analysis may be considered as definite evidence that the Ne and the Nc represent different components.

Probably because a given set of generators activated with a given strength always generates the same electric field and, as a consequence, the same scalp distribution (uniqueness of direct problem solutions), “it is widely assumed that…components are characterized by particular scalp distributions” (Gratton et al., 1989, page 223; see also Picton et al., 2000, for a similar view). Therefore, although it is well known that different sets of generators can produce identical fields at scalp level (unlimited number of inverse problem solutions), one might at least imagine that if two ERPs present different distributions, then their generators are (at least in part) different, and hence correspond to different components. This view is illustrated in Picton et al. (1978) where the authors stated that “Scalp-recorded events with different voltage distributions must derive from different sources…” (page 525), or in Reinhart et al. (2012) where the authors stated that “…different distributions…are traditionally considered to be evidence for different neural sources” (page 2804). In other words, “…two different scalp distributions *cannot* be produced by the same neural generator” (Gratton et al., 1989; page 230; italics by the authors) and these assumptions constitute a logical basis for the functional conclusion of Vocat et al. (2008) presented earlier.

Now, this widely accepted assumption is not necessarily warranted. In a simulation presented by Vidal et al. (2015), a negative and a positive dipole were placed in medial frontal and parietal areas, respectively. It was shown that while the amplitude of the parietal generator was kept constant, a twofold decrease in the amplitude of the frontal one modified its distribution that moved more frontally (Figure 1); this effect occurred because the scalp distribution of the frontal generator became more easily overlapped by the scalp distribution of the parietal one, given that the parietal generator became proportionally stronger than the frontal one (see Figure 2 of Vidal et al., 2015).^4^ In other words, a variation in the intensity of the current issuing, from only one generator among a set of two, can be sufficient to unequivocally modify the scalp potential distribution engendered by this generator. Therefore, scalp voltage distributions are an unreliable tool to firmly identify or separate components, and other methods should be used to clearly separate them in space and time.

**FIGURE 2:**
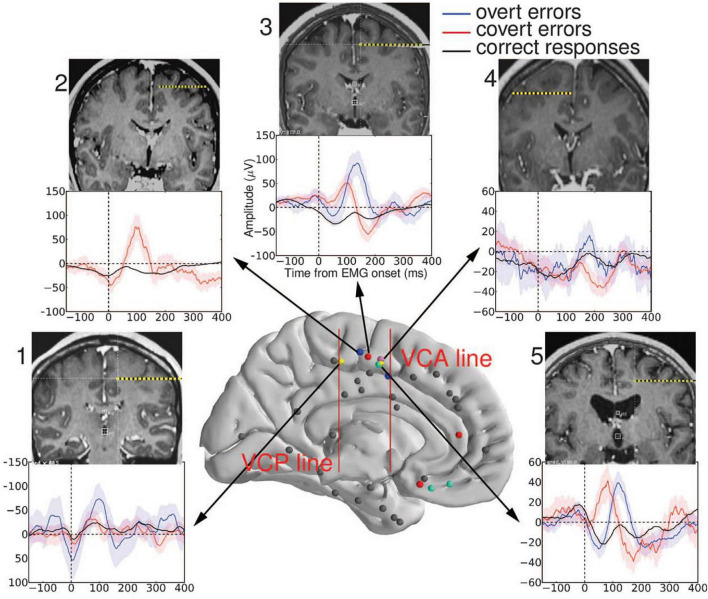
Recording sites in the medial wall. The two red vertical bars represent the vertical commissure anterior (VCA) line and the vertical commissure posterior (VCP) line. A cluster of performance-sensitive sites (colored dots) is located behind the VCA line, in the supplementary motor area (SMA) (caudal cluster). Other performance-sensitive sites are more widespread in the rostral part of the medial prefrontal cortex (electrodes anterior to VCA) and respond later, on errors only. Averaged EMG-locked Local Field Potentials (LFPs) recorded from the SMA are displayed for each participant: The largest LFP is evoked after overt errors (blue); a smaller LFP is evoked after partial errors (red); and an even smaller LFP is evoked on correct responses (black). Colored bands represent between-trials confidence intervals set to 0.05. Individual MRI and CT fusion is also shown for each subject indicating, in coronal view, the trajectory of the performance-sensitive electrode. All these electrodes were clearly located above the calloso-marginal fissure and behind the VCA line (that is, in the SMA) reproduced with permission of the publisher (Science) (adapted from Bonini et al., 2014).

As a consequence, the mere difference in their scalp potential distributions cannot allow one to conclude that the Ne and the Nc have different generators and hence are different components.

To conclude, the main differences usually observed between the Ne and the Nc do not constitute strong conclusive evidence in favor of the idea that the Ne and the Nc are different components.

### Main Similarities

#### Latency

As we have seen earlier, the Nc and the Ne have different latencies, but their latency ranges are similar. The latency of the Nc falls just in between that to the Ne of partial errors and that of the Ne on errors (e.g., Roger et al., 2010; Meckler et al., 2017). Because the latency of the Nc falls in the interval of variations of the Ne, their latencies can be considered as similar. Therefore, the latency of the Nc is perfectly compatible with an interpretation of this wave as small Ne.

#### Possible Similar Amplitudes

Most, if not all, introducing descriptions of the Nc usually refer to its smaller amplitude as compared to the Ne. However, in a handbook chapter, Gehring et al. (2012) indicates that “The correct-response negativity (CRN) is usually smaller than the ERN…” (page 239). The word “usually,” used by the authors, is very important because in certain specific cases, as indicated earlier, this difference vanishes. This is the case in patients with prefrontal focal (Gehring and Knight, 2000) or more diffuse (Hogan et al., 2006) lesions or in patients with schizophrenia (e.g., Ford, 1999; Alain et al., 2002; Mathalon et al., 2002). Now, as also indicated earlier, the Ne and the Nc may also show similar amplitudes in healthy subjects when the required response is unexpected (Meckler et al., 2011). Therefore, although the Ne and the Nc usually have different amplitudes in healthy subjects, this may occur because of the paradigms usually used to study them. It is likely that usual amplitude differences characterize these waves in usual experimental setups only.

#### Scalp Distribution

Scalp potential distributions of the Nc and the Ne are definitely different (Vocat et al., 2008; Meckler et al., 2011; Endrass et al., 2012; Grützmann et al., 2014; Files et al., 2021). However, the scalp distributions of the Ne (whether on errors or partial errors) and of the Nc are undistinguishable after Laplacian transformation (e.g., Vidal et al., 2000; Roger et al., 2010; Meckler et al., 2017). This is very likely because on scalp potentials, the Nc being smaller than the Ne, this wave is more easily overlapped by a large (or several) remote positive component(s), which makes its distribution appear more frontal than it is. As indicated earlier, the Laplacian transformation strongly attenuates volume conduction effects and their consequences in terms of overlapping. The Ne being large, it is not strongly overlapped by remote components on potential maps and the Laplacian transformation just tightens its distribution. Because the Nc is usually smaller, its distribution is much more affected by the same remote components. After Laplacian transformation, overlapping being strongly attenuated, the distribution of the Nc moves back and this wave peaks at the same fronto central topographies, as the Ne. As a consequence, the distributions of the Ne and the Nc appear similar (see Meckler et al., 2017 for an illustration; Vidal et al., 2015 for a more detailed discussion of these overlapping effect on the Nc and their attenuation after Laplacian transformation). Therefore, the Laplacian transformation unmasks the true spatial distribution of the Nc which reveals to be actually similar to the Ne’s. These effects mimic those of the simulation presented in Vidal et al. (2015) where a decreased in amplitude was sufficient to change the distribution of a frontal component in the presence of another unchanging parietal one. In this simulation, the Laplacian transformation also restored the true distribution of the frontal component which revealed, now, to be unaffected by amplitude changes (Figure 1).

Although this observation constitutes a lack of evidence that the generators of the Ne and of the Nc are different, it is not a strong piece of evidence that the generators of these two waves are identical either. Indeed, the application of the Laplacian transformation, although very efficient to separate components in space and time (see Burle et al., 2015), does not solve the inverse problem. The ensemble of generator configurations that could account for Laplacian distributions of the Ne and/or the Nc is certainly reduced as compared to potential distributions, but it remains in principle unlimited. As a consequence, without additional physiological information, the similar (true) distributions of the Ne and the Nc do not fundamentally demonstrate that the Ne and the Nc are the same component.

#### Sensitivity to Performance

It is undisputable that the Ne is sensitive to performance. However, this important functional property is also shared to a certain extent by the Nc.

Ridderinkhof et al. (2003) examined sequential effects on response-locked correct trials ERPs. They compared these ERPs on correct responses whether followed by another correct response or followed by an error. On scalp potentials, these authors evidenced a pronounced positive dip on correct response trials followed by an error, which exhibited a frontocentral distribution. The dip was much smaller on correct response trials followed by another correct response. They named this dip Error-Preceding Positivity (EPP) and considered that because sensitive to performance at the following trial, the EPP revealed an action monitoring mechanism.

Because the Nc is often masked by an overlapping positive component on scalp potentials, and because of the fronto-central distribution of the EPP, Allain et al. (2004a) suspected that the positive dip was due (at least in part) to a reduction of a masked but present underlying Nc on correct trials preceding an error. Put differently, the apparent increased positivity on correct trials preceding an error would be due (at least in part) to a decrease in the amplitude of the Nc buried in the positivity.

In two different experiments, Allain et al. (2004a) reproduced the results of Ridderinkhof et al. (2003) on scalp potential recordings. After Laplacian transformation, a Nc was unmasked on all types of correct trials, but this Nc was clearly smaller on correct trials preceding an error in both experiments.

This demonstrates that the sensitivity to performance is not specific to the Ne. The Nc is also sensitive to performance although not in the same way as the Ne is as Nc reduced amplitude foreshadows errors (on average). In the same line of reasoning as that of Ridderinkhof et al. (2003) for the EPP, one can consider that the sensitivity of the Nc to performance in the next trial suggests that the Nc is also involved in important aspects of action monitoring.

#### Sensitivity to Control or Evaluation

Endrass et al. (2008) showed that not only the Ne (whether on errors or partial errors) was increased in obsessive-compulsive patients as compared to healthy subjects, but also that the Nc was increased. This effect was reduced in patients under medication. Although there are alternative explanations (see Endrass et al., 2008), the effect of illness and of medications in the same direction for the Ne and the Nc might suggest that patients being more involved in evaluating their performance than healthy subjects, this enhancement of the evaluation process equally affects the Ne and the Nc amplitude.

Such an interpretation is in line with data obtained in healthy subjects. As indicated earlier, when healthy subjects are explicitly asked to evaluate their performance at each trial, not only is the amplitude of the Ne increased, but also the Nc one (Grützmann et al., 2014). The fact that evaluation has a similar impact on the Ne and on the Nc suggests that, as it is the case for the Ne, the Nc subserves some kind of action monitoring.

#### Independence From Reafferences

Because the delay between EMG onset and Ne onset is quite short, it has been considered that there was not enough time for reafferent information to reach the brain on time to elicit the Ne (e.g., Gehring et al., 1993). However, the existing 30 ms delay is likely sufficient if one considers that a stimulation of the median nerve takes only 20 ms to reach the brain. To verify whether or not the Ne could actually be independent from reafferences, Allain et al. (2004b) examined whether or not a Ne could be elicited in a patient completely deprived from reafferences from the feet to the chin. A normal Ne was evoked in this patient on errors and partial errors in accordance with the general opinion that the Ne is not dependent on reafferences. Moreover, a normal Nc was also evoked in this patient, indicating that the Nc was reafference-independent as the Ne is.

To conclude, several functional similarities between the Nc and the Ne may argue in favor of the idea that the Ne and the Nc correspond to a unique component. If this were the case, given the definition adopted at the beginning of this article, their generator(s) should be identical. We will examine this question in the following section.

## Tentative Identification of the Generator(s) of the Ne and the Nc

We have seen that the analysis of scalp potential topographies does not allow establishing firm conclusion regarding the nature (same or different) of the generators at the origin of the Nc and the Ne. The Laplacian transformation is very effective at separating generators in space and time because it strongly attenuates volume conduction and its consequential overlapping effects (Law et al., 1993; Burle et al., 2015). However, strictly speaking, the Laplacian transformation does not solve the inverse problem. In other words, if Laplacian scalp distributions of two waves are different, it is highly probable that their generators are also different and that they correspond to two different components. On the contrary, if two waves show the same scalp distribution after Laplacian transformation, it is not completely warranted that they have the same generator(s). Therefore, in the case of the Ne and Nc waves, their similar distributions suggest that they have the same generator(s) but do not allow to definitely adopt this conclusion.

Different strategies have been used to identify the generators of the Ne, the two principal being: (1) using methods to solve the inverse problem or (2) using hemodynamic methods such as functional magnetic resonance imaging (fMRI).

A difficulty when using inversion methods is that their reliability and their results may depend on the underlying assumptions of the model needed to obtain a single solution. For example, Roger et al. (2010), using Independent Component Analysis (ICA; Jung et al., 2001) to separate the Ne from other components, applied two methods of inversion to localize the Ne “component” (the term “component” here is used in its ICA statistical acceptation). First, the authors used the “dipfit” module of EEGLAB. The median position of individual dipoles was unequivocally found in the Rostral Cingulate Zone (RCZ: Picard and Strick, 1996, 2001) at coordinates that correspond to the anterior cingulate cortex (ACC). Secondly, the authors used the sLORETA distributed source localization method (Pascual-Marqui, 2002). They identified a broadly distributed area corresponding roughly to the RCZ encompassing the ACC but also the SMAs with maximal statistical significance lying more superficially than the ACC, in the supplementary motor area (SMA)/pre-SMA (see Figure 7 of Roger et al., 2010), which constitutes a notable difference with the solution obtained with the “dipfit” method.

Moreover, few studies have tried to localize the Ne *and* the Nc. With both of the two localization methods used by Roger et al. (2010), these authors found a same generator for the Nc and the Ne (whether on errors or partial errors). However, the RCZ being quite large and the distributed source also extending to the pre-SMA/SMA, as pointed out by the authors, these results, although suggesting a unique generator for both waves, did not constitute an undisputable demonstration.

Hemodynamic methods, although very useful, lack the temporal resolution that might be necessary to unequivocally identify the generator(s) of the Ne and the Nc. To circumvent this difficulty, fMRI source identifications of the Ne have been attempted on the basis of single trial Ne amplitude analysis (Debener et al., 2005; Iannaccone et al., 2015).

Debener et al. (2005) first separated the Ne from other components with ICA and, secondarily, used amplitude estimations of single-trial Ne to predict the fMRI BOLD signal. This fMRI analysis showed that the BOLD response specifically correlated with the amplitude of the Ne in a RCZ cluster extending from the dorsal ACC (dACC) to the pre-SMA (but also area 8), with a center of gravity lying just at the border between these two structures (coordinates: 0, 17, 42). Iannaccone et al. (2015) used a similar strategy without resorting to ICA, however. Their fMRI analysis also showed that the BOLD response specifically correlated with the amplitude of the Ne in a large cluster comprising not only the dACC but also extending to the pre-SMA. Therefore, even with EEG-informed fMRI analysis, the set of candidate structures possibly generating the Ne remains quite large, comprising at least the dACC and the SMA, while, to our knowledge, no tentative has been done to identify the generator(s) of the Nc with EEG-informed fMRI analysis and compare them to those of the Ne.

Field Potentials “seed the electroencephalograms (EEG) that are recorded from outside the brain” (Herreras, 2016, page 1), their signal to noise ratio is much better than that of EEG (they are not attenuated by the highly resistive skull) and they are not blurred by currents diffusion through the resistive skull. This is likely why some authors resorted to direct intracerebral recordings in monkeys or in humans to localize the generators of the Nc or the Ne. However, a word of caution is in order because the interpretation of Local Field Potentials (LFPs) is not necessarily straightforward. Variations in LFPs amplitude is not always due to variations in the intensity of the neuronal currents which generate them. Because of the quasi-dipolar nature of the main current generators at the neuronal level, geometric effects within and between neurons may strongly affect the amplitude and time course of recordable LFPs due to possible spatial cancelation effects (see Herreras, 2016, for a detailed and comprehensive review regarding these geometric effects). Even at the individual neuron level, these spatial cancelation effects of geometric origin may vary between experimental conditions depending on the pattern of synaptic excitatory/inhibitory influences received from different afferent pathways at different neuronal locations (Herreras, 2016). Moreover, of course, because LFPs are not immune to volume conduction, the fact that LFPs are recorded in a given structure does not warrant that they are generated by this very structure, unless specific observations are made to assert this point. Nevertheless, provided that they are interpreted with the appropriate discernment, taking into account the preceding reservations, LFPs may constitute a valuable tool to examine the sensitivity of different structures to experimental manipulations.

Therefore, in the following sections, we will examine results mainly yielded by this approach.

### Intracerebral Recordings in Monkeys

Before presenting the data obtained with intracerebral recordings in monkeys, it is necessary to demonstrate that a Ne is present on errors in the scalp-recorded EEG of monkeys and that its characteristics are similar to those observed in humans.

On scalp-recorded potentials, Reinhart et al. (2012) reported in human subjects realizing a countermanding task the presence of a Ne for errors not only realized with the hands but also for errors realized with the eyes in the same countermanding task. On scalp potential recordings, Godlove et al. (2011) clearly showed the existence of a Ne on saccade errors in monkeys performing a countermanding task (see also Sajad et al., 2019). Now, in a follow up study, Reinhart et al. (2012) showed that the characteristics of the Ne on saccade errors were extremely similar in monkeys and in humans. Moreover, a clear Nc was also evoked on correct saccades (Sajad et al., 2019; see also Figure 2 of Godlove et al., 2011). Therefore, studying intracerebral recordings in monkeys to identify the generators of the Ne and the Nc seems relevant.

When monkeys realized a countermanding task executed with the arm, Scangos et al. (2013) found that the properties of the evaluative cells that monitor the outcome of actions in the SMA/pre-SMA (among which are error-responding cells) were extremely similar to those of the supplementary eye fields (SEF) in a similar countermanding task executed with the eyes. The authors considered that “the strong similarity in the evaluative signals, found in pre-SMA, SMA, and SEF is … quite remarkable…” (page 1,938). Then, it seems reasonable, in the following, to consider the activities related to action monitoring observed in the SEF or from surface electrodes for eye movements as the analog of the activities related to action monitoring observed for segmental movements in the SMA/pre(SMA) or from surface electrodes.

Emeric et al. (2008) recorded LFPs in the ACC of monkeys performing a saccade countermanding task and described error-related LFPs which properties looked similar to those recorded at scalp level by Godlove et al. (2011) or to those of humans performing a similar task (Reinhart et al., 2012). In a follow-up study, Emeric et al. (2010) recorded the LFPs from the SEF of monkeys performing the same task. These authors described “A small CRN…following saccades on no stop signal trials, but following error saccades a more pronounced negative polarization…” (Emeric et al., 2010, page 1,529) in latency ranges corresponding to those of the Nc and the Ne.

An important point was raised by Emeric et al. (2008). Because currents are volume-conducted through brains tissues, the presence of error-related LFPs in the ACC and/or the SEF does not warrant that these structures are the generators of theses LFPs. For example, as noted by the authors as regards ACC error-related LFPs, these potentials could well be generated in the ventral bank of the ACC or in the SEF or both (the same argument, of course, holds for error-related LFPs recorded in the SEF).

In light of the analysis performed later by Emeric et al. (2010), this possibility appears quite unlikely though. The onset distributions of error-related LFPs recorded in the ACC were delayed by about 50 ms as compared to the onset distributions of error-related LFPs recorded in the SEF. If LFPs recorded in one of these structures were volume-conducted from the other one, their time courses would be identical. Now, one cannot exclude, on this argument only, that these activities have been volume-conducted from other neighboring structure(s).

To evaluate this possibility, the authors compared the time course of error-related LFPs recorded in the SEF (by Emeric et al., 2010) and the ACC (by Emeric et al., 2008) with error-related spike rate modulations observed in the ACC (by Stuphorn et al., 2000) and in the SEF (by Ito et al., 2003). The distributions of the onsets of error-related LFPs and error-related spike rate modulations revealed to be strikingly similar (almost undistinguishable from one another in the SEF: Figure 7 of Emeric et al., 2010). This suggests that they are very closely related functionally. Now, because of their quasi quadrupolar nature (Pernier, 2007), the fields generated by spiking activities decrease more steeply with distance than do the fields generated by the quasi-dipolar post-synaptic activities which are the main contributors (Buzsáki et al., 2012) to the LFPs. Therefore, it can be safely concluded that the error-related spiking modulations recorded in the ACC and the SEF are generated in these very structures. If these are closely related to the corresponding recorded LFPs, as suggest their almost identical temporal distributions, the error-related LFPs recorded in the ACC and in the SEF did arise from these structures.

Finally, Sajad et al. (2019) showed that, on saccade errors, variations of error-related spiking of SEF cells (but no other spiking) in layers 2/3 (but not in layers 5/6) predict variations in the amplitude of the Ne recorded on the scalp of the monkeys. Given the close temporal relationships between the error-related spiking of the SEF and error-related LFPs of this same structure (Emeric et al., 2010), the results of Sajad et al. (2019) demonstrate that the SEF are one generator of the surface Ne on saccade errors.

What about the Nc? As indicated earlier, a Nc-like LFP has been described in the SEF (Emeric et al., 2010). Sajad et al. (2019) also showed that, on correct saccades, variations of the spiking of error-sensitive cells of SEF layers 2/3 (which fire less on correct saccades than on errors but still fire with a similar time course) predict variations in the amplitude of the Nc recorded on the scalp. Such a relationship is also absent for neurons of layers 5/6. Therefore, in the same line of reasoning as that adopted for errors, these results demonstrate that the SEF are one generator of the surface Nc on correct saccades (Sajad et al., 2019).

Finally, could it be that on a countermanding saccadic task, the SEF generators of the Ne and possibly of the Nc are different? The “…association between SEF L2/3 spike rate and EEG on both error and correct trials argues against this possibility” (page 271).

To sum up, evidence based on intracerebral recordings in monkeys indicate that for saccadic movements, (1) the SEF *and* the ACC do generate error-related LFPs, (2) SEF error-related LFPs precede error-related ACC LFPs by about 50 ms, (3) Comparison of variations of SEF layers 2/3 error-related spiking neurons with scalp recordings on errors indicate that the SEF are (at least one of) the generator(s) of the Ne, (4) the SEF generate correct-related LFPs, and (5) comparison of variations of SEF layers 2/3 error-related spiking neurons on correct saccades with scalp recordings on correct trials indicate that the SEF are (at least one of) the generator(s) of the Nc.

### Intracerebral Recordings in Humans

Intracerebral data are scarcer in humans than in monkeys, but provide valuable information.

Brázdil et al. (2002, 2005) recorded intracerebral LFPs from humans performing a Go/Nogo task involving manual responses to identify the possible generators of the Ne. These authors convincingly showed that the LFPs recorded in the ACC and the pre-SMA were consistently sensitive to errors. These authors also showed that several other structures were actually responding to errors, which demonstrates that a large network of brain structures is involved in (or at least sensitive to) performance monitoring processes. However, it is not clear whether or not these other structures could actually contribute to the observed distribution of the Ne on scalp potentials and on Laplacians.

Bonini et al. (2014) also recorded LFPs from human participants involved in a two- choice between-hand Simon task (Simon, 1990 for a review), known to generate errors. The authors found that the EMG-locked LFPs recorded in the SMA-proper (SMAp) were strongly sensitive to performance, being large on full-blown errors, smaller on partial errors, and even smaller but clearly present on correct responses (Figure 2). These LFPs started shortly after EMG onset and peaked in the latency ranges of the Ne and the Nc. Steep voltage gradients excluded the possibility that these recorded LFPs would correspond to volume conducted potentials generated in other structures. Moreover, in one participant, two recording contacts, placed in the upper and lower parts of the SMAp, recorded Ne-like and Nc-like LFPs. But these activities were mirror images of each other. Such polarity reversal is the typical signature of a dipole seen from two opposite sites, demonstrating that in this participant, the generators of the Ne-like and Nc-like LFPs were located between these two contacts, that is, in the SMAp (Bonini et al., 2014, see Supplementary Material). These points are important to consider as indicated earlier. Therefore, in humans, Ne-like LFPs are generated on errors and partial errors and Nc-like LFPs are generated on correct responses by the SMAp, which suggests that the SMAp is a common generator of the Ne and the Nc when humans perform manual responses, as it is the case for the SEF in monkeys performing saccade responses. More anterior structures which could be explored in two subjects were also performance-sensitive: the pregenual anterior cingulate cortex and the medial orbitofrontal cortex (but see also Brázdil et al., 2002, 2005 for performance-sensitive LFPs in the latter). However, two main differences were observed between these anterior regions and SMAp activities: (1) the activities of the anterior regions were delayed (as compared to those of the SMAp) with a caudo rostral latency gradient (between 200 and 350 ms post-EMG onset) and (2) they only exhibited error-related LFPs but no correct-related LFPs. Given their deep and very anterior position and because of their late latencies, it is very doubtful that these activities would contribute to the Ne. However, they certainly participate (or are sensitive) to action monitoring processes and seem to classify correct and error trials in an all or none fashion. Finally, because the Ne could be identified in most of single trials in intracerebral recordings, a positive correlation could be evidenced on a trial-by-trial basis between the amplitudes and, more importantly, between the latencies of the error-related LFPs generated in the SMAp and those generated in rostral performance-sensitive structures. Moreover, in all trials, the SMAp error-related LFPs preceded those of the recorded anterior mid prefrontal structures, indicating a leading role of the SMAp during action monitoring operations, with a hierarchy following a caudo rostral gradient, the SMAp being upstream.

More recently, Fu et al. (2019) recorded LFPs and unit activities in the pre-SMA and the ACC in humans involved in a manual variant of the Stroop (Stroop, 1935) naming color task. The authors identified error-sensitive neurons in both pre-SMA *and* ACC along with error-related LFPs in both structures. The error-related neuronal responses and the error-related LFPs appeared in the pre-SMA first, while error-related neuronal responses and error-related LFPs showed up later in the ACC, with a delay of about 50 ms (55 ms for spiking responses and 40 ms for LFPs peaks). These delays are extremely similar to those reported by Emeric et al. (2010) between SEF error-related LFPs and ACC error-related LFPs in monkeys (about 50 ms) performing a saccade countermanding task. Finally, on a trial-by-trial basis, the amplitude and the latency of the error-related LFPs in the ACC were shown to be positively correlated to the amplitude and the latency of the preceding error-related LFPs of the pre-SMA, confirming the leading role of the pre-SMA on the ACC in a caudo-rostral hierarchy of performance monitoring processes. Finally, the amplitude of error-related LFPs was larger in the pre-SMA than in the ACC.

What about the Nc? Although their scalp recordings did show a Nc (Fu et al., 2019, Supplementary Material), the authors did not mention correct-related LFPs, and their Figure 5B representing an example of event-related LFPs recorded from the ACC did not show any sign of the presence of a Nc either. Contrary to scalp potential recordings, it is almost impossible that a Nc, if present, would be masked by some kind of overlapping effect. Therefore, it must be (provisionally) concluded that the ACC does not generate correct-related LFPs.

To sum up, evidence based on intracerebral recordings in humans performing a RT task with the hands indicate that (1) a large brain network is involved in (or at least sensitive to) performance monitoring processes, (2) the supplementary motor area proper/pre-supplementary motor area (SMAp/pre-SMA) *and* the ACC do generate error-related LFPs that could be at the origin of the scalp-recorded Ne, (3) SMAp/pre-SMA error-related LFPs precede error-related LFPs in the ACC and in more rostral medial prefrontal structures, (4) the latencies of error-related LFPs of caudal structures foreshadow those of error-related LFPs of more rostral structures, and (5) the SMAp generates correct-related LFPs.

### Functional Consequences of Intracerebral Recordings Data

First, there are very strong similarities between the intracerebral data obtained in monkeys and those recorded in humans, among which (1) the existence of error-related LFPs in the SEF (monkeys) or the SMAp/pre-SMA (humans) *and* in the ACC, (2) the precedence of SEF or SMAp/preSMA responses on ACC responses which appear similarly delayed in monkeys and in humans, and (3) the presence of correct-related LFPs in the SEF of monkeys and in the SMAp of humans. These major similarities allow considering that the observations made with intracerebral recordings in monkeys likely hold in humans. This is the option taken in the following.

#### The Nc and the Ne Share a Main Common Generator in the Supplementary Eye Fields SMA-Proper/Pre-SMA

Given the temporal relationships existing between error-related LFPs and error-related spike modulations in the SEF (Emeric et al., 2010), on the one hand, and the fact that error- *and*, correct-related spike modulations of the same neurons in layers 2/3 predict on a trial by trial basis the amplitude of the Ne *and* of the Nc at scalp level (Sajad et al., 2019), on the other hand, it can be concluded that the error and correct-related LFPs in the SEF are a main generator of the Ne and the Nc when movements are performed with the eyes. If, as argued earlier, we admit, along with Sajad et al. (2019), that the activities related to action monitoring observed in the SEF for eye movements are the analog of the activities related to action monitoring observed in the SMAp/pre-SMA for segmental movements, then the error-related LFPs generated in the SMAp/pre-SMA (Bonini et al., 2014; Fu et al., 2019) and the correct-related LFPs generated (at least) in the SMAp (Bonini et al., 2014), are the main generators of the Ne and the Nc, respectively.

It must therefore be concluded that the Nc and the Ne have a main common generator.

#### Contribution of the Anterior Cingulate Cortex to the Ne or the Nc

No correct-related LFPs seems to develop in the ACC (Figure 5B of Fu et al., 2019), although a clear Nc shows up on scalp recordings (Fu et al., 2019, Supplementary Material). In this event, then the sensitivity of the ACC to performance would be in an all or none fashion as it is the case for more rostral pregenual or medial orbitofrontal areas (Bonini et al., 2014). The SMAp/pre-SMA would react first to performance in a graded manner, being upstream in a caudo-rostral hierarchy of performance monitoring processes and, at its output or later on downstream, a thresholding process would allow classifying performance in an all or none manner in the ACC and in more rostral structures, with the ACC possibly being in second position in this caudo-rostral hierarchy.

What is the contribution of the ACC to the Ne? On the basis of available data, it is not possible to draw a firm conclusion. The ACC is situated deeper that the SMAp/pre-SMA which are quite superficial, and the strength of the ACC error-related LFPs is weaker than the strength of pre-SMA error-related LFPs (Fu et al., 2019). At face value, these two observations suggest that the contribution of the ACC to the Ne should be much smaller than that of the SMAp/pre-SMA or even negligible.

However, such a conclusion should be taken with appropriate skepticism because the influence of a given generator at scalp level not only depends on its strength but also on its orientation. Therefore, although the strength of the ACC generator is smaller, it could well be that its orientation is more optimal than that of the SMA/pre-SMA generator to produce a significant contribution to the Ne at scalp level.

It is clear that all things being equal, surface potentials are less sensitive to deep than to shallow generators and surface Laplacians are even less (Pernier et al., 1988). It is possible, though, that the “electric distance” of the ACC generator is not that far from the scalp. When issuing from the medial wall or the cingulate sulcus, currents may encounter a wall of higher resistance than that of brain tissues and cerebrospinal fluid: the *falx cerebri*. Given its sagittal orientation, this more resistive structure might guide currents in a preferential vertical plane and make the access of the ACC currents to the surface easier.

Then it is quite difficult to assess, on the sole basis of its depth and of the intensity of its LFPs, which is the possible contribution of the ACC to the Ne.

Finally, given the delay observed between SEF or pre-SMA error-related responses and ACC error-related responses, it seems clear that if the ACC significantly contributes to the Ne, it contributes to its latest part only, with the initial part of the Ne being generated by the SEF, SMAp/pre-SMA.

Because the Laplacian transformation reduces overlapping effects in time and space, it could be expected that its application would separate the activities issuing from the SMAp/pre-SMA from those issuing from the ACC. This is not warranted, though, in this particular case because the separation power of the Laplacian transformation is probably not strong enough to isolate from the scalp surface the activities generated by two almost superimposed structures. Unfortunately, statistical methods such as ICA would also likely fail to separate the activities of these structures for the same reasons. This may also be why, as indicated earlier, non-invasive tentative localizations of the Ne generator point around the RCZ/ACC and SMAp/pre-SMA without a firm discrimination between these two regions.

#### Waves and Components

If the ACC does contribute to the Ne, then the Ne is a wave but not a component, for two independent reasons: (1) *two different structures* activated with (2) *two different time courses* would generate the Ne. If the Ne is a compound wave made of two components overlapping in time and space on scalp potentials or on scalp Laplacians, “…then it probably manifests multiple computations and representations. If so, then no single exclusive theory of the ERN is possible.” (Sajad et al., 2019, page 271). It could well be that in usual paradigms where errors are rare that the SEF SMAp/pre-SMA responds first to correctness, quite weakly on correct responses, strongly on errors, and in between on partial errors. These (early) evoked activities when recorded at the scalp would correspond to a unique component, sensitive to correctness in a graded manner. Later on, after a thresholding process, another component would arise from the ACC on errors only the ACC being sensitive to correctness in an all or none manner. This component could be the same as the one evoked by feedback error signal, namely, the Feedback-Related Negativity (FRN: Miltner et al., 1997), since it has been established that the ACC does generate feedback-related error LFPs (Emeric et al., 2008), whereas the SEF do not (Emeric et al., 2010).

If the absence of correct-related LFPs in the ACC is confirmed, then the Nc *is* a component clearly involved in action monitoring, and this component is the same (although smaller) as the first component (in time) of the Ne wave, because generated with the *same time course* by the *same structure* and even the *same neurons* (Sajad et al., 2019).

What about the Ne on partial errors which, to our knowledge, has been studied only with intracerebral recordings by Bonini et al. (2014)? The case of the partial error Ne is likely even more complex than that of the Ne on full-blown errors. Partial error-related LFPs are generated in the SMA (Bonini et al., 2014). If the ACC would account for the last part of the Ne on full-blown errors, could we extrapolate that this should also hold for the Ne of partial errors? Maybe not.

The RT of a correct response following an error is longer than the RT of a correct response following a correct response (Laming, 1979). Allain et al. (2009) showed that this so-called post-error slowing (PES) is not specific to errors. There is also a post (partial)-error slowing although much smaller than the post (full-blown)-error slowing. However, it has been demonstrated recently that the post (partial)-error slowing effect is not evenly distributed among the population of partial errors (Ficarella et al., 2019). Most partial errors are consciously undetected (Nieuwenhuis et al., 2001; Rochet et al., 2014) but only consciously detected partial errors, those which generate the largest Ne (Ficarella et al., 2019), give rise to a post (partial)-error slowing (Ficarella et al., 2019). Fu et al. (2019) examined the relationship between error-related pre-SMA and ACC activities and PES. The authors investigated if neural synchrony would predict PES, on a trial-by-trial basis, with neural synchrony being defined as the extent to which error-related spike rates co-vary with the amplitude of error-related LFPs. In the ACC, but not in the pre-SMA, neural synchrony predicted PES: the larger the correlation between error-related spike rate and error-related LFPs, the larger the PES. Therefore, it seems that to generate a PES, error signals must at least reach the ACC (from the SMAp/pre-SMA). If there is no PES, it is possible that such error signals do not reach the ACC. It could therefore well be that the majority of unconsciously detected partial errors generate partial error-related LFPs in the SMAp/pre-SMA, but not in the ACC. On the contrary, consciously detected partial errors could generate partial error-related LFPs in the SMAp/pre-SMA *and* in the ACC. In other words, the Ne of unconscious partial errors would be a component while the Ne of conscious partial errors would be a wave made of two distinct components.

## Provisional Conclusion and Their Possible Consequences

From what precedes, it can be firmly concluded that the Ne (in full-blown and partial errors) and the Nc share a main common generator situated in the SEF SMA/pre-SMA.

The exact contribution of ACC error-related LFPs to the Ne, because of their lower intensity and because of their deep position, needs to be more documented, possibly by studying the relationships between intracerebral error-related ACC activities (whether spiking or LFPs) and the Ne. The question should be more difficult for the ACC than it has been for the SEF, SMAp/pre-SMA: being downstream to these structures, any correlation between ACC error-related activities and surface Ne amplitudes could result from an upstream influence of the SEF SMAp/pre-SMA on the ACC.

If the contribution of the ACC to the surface Ne is negligible (which is far from being warranted, as indicated earlier), then the Ne and the Nc are a same component which varies in amplitude with correctness. This would put strong constraints on the models accounting for the Ne because they should also account for the Nc and its sensitivity to various experimental conditions. Moreover, the subtracting procedure for modeling, for localization or for interpretation would also be definitely invalid.

However, let us provisionally assume, as our present best bet, that when errors are rare, (1) the ACC does contribute to the scalp-recorded Ne, (2) the SEF SMAp/pre-SMA is the main generator of the Nc (and the Ne on unconscious partial errors), and of the early part of the Ne on errors (and conscious partial errors), but not of the FRN (Emeric et al., 2010). As a consequence, the Nc, the Ne on unconscious partial errors, and the early part of the Ne on errors or conscious partial errors would correspond to a same component, (3) that the ACC is the main generator of the latest part of the Ne on errors or conscious partial errors and of the FRN (Emeric et al., 2008). Therefore, the latest part of the Ne on errors or conscious partial errors, and the FRN might correspond to a same component.

In such a view, when errors are rare, ACC would always respond to errors. In case of “action” errors, SEF SMAp/pre-SMA would generate a large internal signal which, if it crosses a set threshold, would be sent to the ACC that will signal, in all or none fashion, the presence of an error. When information concerning the error is external (feedback from the environment), this would directly activates the ACC and generate the FRN.

In other words, when errors are rare, the ACC would respond on errors to the first (internal or external) signals conveying error information, particularly, internal signals being generated by SEF SMAp/pre-SMA.

This rough sketch of the possible contribution of SEF SMAp/pre-SMA to error processing would easily explain why EEG-informed fMRI analysis (Debener et al., 2005; Iannaccone et al., 2015) fail to distinguish between SMAp/pre-SMA and ACC Ne-related hemodynamic responses. If the early part of the Ne is generated by SMAp/pre-SMA while its latest part is generated by the ACC, then the resulting activation is necessarily a mixture of the sequential activations of each of these structures which cannot be disentangled, because they are modeled as a single phenomenon.

Moreover, subtracting correct-evoked from error-evoked responses would be physiologically meaningless and misleading whether for localization, interpretation, or modeling.

Finally, to our knowledge, none of the aforementioned models can accommodate a two-component account of the Ne in their present form. It is possible, however, that some modifications of these models could solve this problem. Nevertheless, letting the two-component issue apart, let us examine how these models would perform under our preceding assumptions.

The mismatch theory easily accounts for the Ne being usually smaller on partial errors than on full blown errors because as partial errors are “less erroneous,” the mismatch should also be smaller. Now, this theory cannot account for the existence of a Nc if this component is the same (although smaller) than the initial part of the Ne; moreover from what precedes, considering that a “true” Ne would correspond to the difference between the Ne and the Nc would appear to be physiologically ill-founded.

The conflict model in its present form, although assuming possible correct-related potentials, cannot accommodate a same latency range for these potentials and the Ne. It should be examined whether some adaptations of the model would be compatible with the Nc and the Ne developing in the same latency range. Although the observed relationships between the time needed to correct partial errors, on the one hand, and the amplitude of the Ne on partial error trials, on the other hand, violate the predictions of the model in its present form (Burle et al., 2008), the conflict model clearly predicts the existence, in average, of a Ne evoked by partial errors, with smaller amplitude than on full-blown errors.

The reinforcement learning model is hardly compatible with the existence of a Nc if this component is the same (although smaller) as the initial part of the Ne because a core assumption of the model is that the Ne is generated on errors only. If the early part of the Ne and the Nc correspond to a same component, this core assumption is invalidated. It is not clear as to whether the model makes any prediction regarding partial errors given that on partial error trials, unless partial errors are considered as responses (which strictly speaking they are not) preceding another (correct) response in the same trial.

The predicted response outcome model and the reward value and prediction model both predict that the medial prefrontal frontal cortex generates response evaluation signals on erroneous *and* on correct trials in the same latency ranges. The models also predict that when errors are rare, these signals should be larger on errors than on correct responses. Therefore, these two models nicely fit with the idea that the Nc and (at least a part of) the Ne do correspond to a same physiological or computational process, or put differently, that they correspond to a same component. Moreover, although the reward value and prediction model does not account for the elicitation of a Ne on partial errors yet, the authors indicate that a process for partial error detection might be included in the model. Whether modified versions of the response outcome model might also account for the elicitation of the Ne needs to be specified.

The conclusion that the Ne wave on errors does not correspond to a single component and share a common generator with the Nc complexifies the EEG study of action monitoring and may seem quite demoralizing for those who rely on EEG studies for unraveling (psycho) physiological performance monitoring processes. However, once the generators of the Ne and the Nc will be perfectly characterized, for example, through future intracerebral recordings, and provided that the Ne/Nc are correctly separated in space and time from other components, it might be conceivable to solve the inverse problem by constraining the number and the position of the Ne generators on the basis of previous accurate characterizations. In this case, it might become possible to work not in the space of the sensors but in the space of the generators, which time course and sensitivity to experimental manipulations might be studied separately, thanks to EEG recordings.

## Author Contributions

All authors listed have made a substantial, direct, and intellectual contribution to the work, and approved it for publication.

## Conflict of Interest

The authors declare that the research was conducted in the absence of any commercial or financial relationships that could be construed as a potential conflict of interest.

## Publisher’s Note

All claims expressed in this article are solely those of the authors and do not necessarily represent those of their affiliated organizations, or those of the publisher, the editors and the reviewers. Any product that may be evaluated in this article, or claim that may be made by its manufacturer, is not guaranteed or endorsed by the publisher.

## Footnotes

1 The Laplacian transformation, acting as a high-pass spatial filter reduces volume conduction effects and, as a consequence, overlapping effects in space (Babiloni et al., 2001; Nuñez and Srinivasan, 2006; Kayser and Tenke, 2015) and secondarily in time (Law et al., 1993; Burle et al., 2015).

2 Moreover, even though the Ne and the Nc would correspond to different components, it is also to be assumed that experimental manipulations that might affect the Ne let the Nc unaffected, a condition which is empirically not always met.

3 It is noteworthy that in line with the predictions of the model, neurons can be found (in medial prefrontal cortex of monkeys: ACC, Anterior Cingulate Cortex) which encode not only outcome values (Amiez et al., 2005, 2006) but also prediction errors (Matsumoto et al., 2007; Kennerley et al., 2011) which suggest, as the model does, that the medial prefrontal cortex might be sensitive to outcome unexpectedness, whether response outcome is worse or better than expected. This idea is supported by fMRI data in humans where, in a gambling task, the error effect is reversed when losses are more likely than gains (Jessup et al., 2010). Note, however, that these activities are essentially feedback-related.

4 However, Laplacian-transformation of the same data set conserved unchanged the scalp distribution of the frontal component, because reducing overlapping effects.
